# Adaptation and validation of a tool for assessing food knowledge based on the Nova classification for the Brazilian context

**DOI:** 10.1590/S2237-96222024v34e20240335.en

**Published:** 2025-05-02

**Authors:** Kamila Tiemann Gabe, Gilberto Bassetto, Patricia Constante Jaime

**Affiliations:** 1 Universidade de São Paulo, Núcleo de Pesquisas Epidemiológicas em Nutrição e Saúde, São Paulo, SP, Brazil; 2 Universidade de São Paulo, Faculdade de Saúde Pública, Departamento de Nutrição, São Paulo, SP, Brazil

**Keywords:** Dietary Guidelines, Food and Nutrition Education, Health Literacy, Nutrition and Food Programs and Policies, Validation Studies, Guías alimentarias, Educación alimentaria y nutricional, Alfabetización en salud, Programas y políticas de nutrición y alimentación, Estudios de validación

## Abstract

**Objectives:**

To adapt and validate a tool to measure the level of food knowledge based on the Nova classification for the Brazilian context.

**Methods:**

A tool developed by Canadian researchers was adapted for Brazil. In this tool, respondents assign healthiness scores to 12 images of foods with different levels of industrial processing according to the Nova classification – unprocessed and minimally processed, processed and ultra-processed. Total score is computed by comparing scores assigned to foods from different groups, and range from 0 to 8. The Brazilian version, named Nova-Conhecimento, was evaluated by experts and submitted to pre-tests with potential users. Discriminant validity was assessed by comparing scores of undergraduate students of nutrition and undergraduate students in education-related fields. Convergent validity was assessed by testing the association between the knowledge score and the consumption of ultra-processed foods in a subsample of the NutriNet Brazil cohort (n=1,245).

**Results:**

Nutrition students had higher scores than education students (6.7 vs. 5.3; p-value<0.001). Each point in the knowledge score was associated with a reduction of 1.03 percentage points in the contribution of ultra-processed foods to the diet (p-value<0.001).

**Conclusion:**

The Nova-Conhecimento tool demonstrated validity and can contribute to food and nutritional surveillance activities based on the the *Dietary Guidelines for the Brazilian Population*.

## Resumen

**Objetivos:**

Adaptar y validar, para el contexto brasileño, una herramienta para medir el nivel de conocimiento sobre alimentación según la clasificación Nova.

**Métodos:**

Se adaptó para Brasil una herramienta desarrollada por investigadores canadienses, en la cual se asignan puntuaciones de salubridad a 12 imágenes de alimentos con diferentes niveles de procesamiento industrial según la clasificación Nova: alimentos in natura y mínimamente procesados, procesados y ultraprocesados. Las puntuaciones se calculan comparando las evaluaciones asignadas a alimentos de diferentes grupos, con un rango de 0 a 8. La versión brasileña, denominada Nova-Conocimiento, fue evaluada por especialistas y sometida a pruebas piloto con usuarios potenciales. Se evaluó su validez discriminante comparando las puntuaciones de estudiantes de nutrición (n=76) con las de estudiantes de licenciaturas en otras áreas (n=99). La validez convergente se analizó probando la asociación entre la puntuación de conocimiento y el consumo de ultraprocesados en una submuestra de la cohorte NutriNet Brasil (n=1.245).

**Resultados:**

Los estudiantes de nutrición obtuvieron puntuaciones superiores a las de los estudiantes de otras licenciaturas (6,7 frente a 5,3; p-valor<0,001). Cada punto adicional en la puntuación de conocimiento se asoció con una reducción de 1,03 puntos porcentuales en la proporción de ultraprocesados en la dieta (p-valor<0,001).

**Conclusión:**

La herramienta Nova-Conocimiento demostró ser válida y puede contribuir a las actividades de vigilancia alimentaria y nutricional en el marco de la *Guía Alimentaria para la Población Brasileña*.

## Introduction

One of the primary functions of a food-based dietary guideline is to serve as an official and reliable source of information on healthy eating for the population of each country (1). The second edition of the *Dietary Guidelines for the Brazilian Population*, published in 2014 by the Ministry of Health, introduced for the first time, recommendations entirely based on the level of food processing, summarized by the golden rule “always prefer fresh and minimally processed foods and their culinary preparations over ultra-processed foods .”(2).

This central message was based on data indicating a trend among the Brazilian population to replace the consumption of traditional foods, such as rice and beans, with the consumption of ultra-processed foods. (3). This message has proven increasingly relevant, given the growing body of evidence on the negative health impacts of higher consumption of these foods (4). In order to achieve a reduction in the consumption of ultra-processed foods, it is essential to promote the population’s knowledge of how to identify these foods and recognize them as unhealthy, thereby enabling individuals to make autonomous healthy eating choices.

Since its publication, various actions have been undertaken to disseminate the recommendations of the Guidelines, such as the publication of booklets, videos and brochures, the training of health professionals and the inclusion of messages in textbooks (5). Nevertheless, the ability of the Brazilian population to distinguish foods as more or less healthy, considering their degree of processing, remains under-researched, limiting the possibility of assessing the impact of the Guidelines. This gap is partly due to the lack of validated tools that allow for such measurement.

A study that reviewed tools for promoting healthy eating and assessing adherence to the Guidelines found two instruments developed in Brazil to evaluate individuals’ knowledge based on the Guidelines’ recommendations. Of these two instruments, one is designed for health professionals and the other for children, and neither of them is focused on measuring the ability to recognize ultra-processed foods (6). Recently, Canadian researchers developed a tool to measure food knowledge based on Nova, the food classification system according to degree of processing that introduced the concept of ultra-processed foods and is adopted in the Guidelines (7).

The food processing knowledge tool (FoodProK) is used to assess individuals’ ability to recognize ultra-processed foods as less healthy than foods in other categories of the Nova classification by assigning healthfulness scores to various foods (7). A score is computed based on the comparison of scores assigned to foods, according to groups of the Nova classification. The objective of this study was to adapt and validate this tool for the Brazilian context, in order to measure the level of knowledge of food according to the Nova classification.

## Method

The adaptation and validation of a tool to assess food knowledge based on the degree of food processing were conducted for the Brazilian context.

In the FoodProK, the tool used as a model for developing the Brazilian tool, respondents are asked to rate, on a scale of 1 to 10, how healthy they consider a sequence of 12 food items distributed into four categories – fruits, meats, dairy products and cereals. The foods are presented individually in random order; packaged items are accompanied by a nutrition facts label and an ingredient list. Within each of the four categories, one food item from each of the following Nova food classification groups is presented: unprocessed and minimally processed foods (G1), processed foods (G3) and ultra-processed foods (G4). Processed culinary ingredients (G2) are not included in the tool, as they are not usually consumed alone, but as part of culinary preparations. To assess knowledge, it is expected that, within each food category, individuals will assign the highest rating to food belonging to G1; an intermediate rating to the food belonging to G3; and the lowest rating to the food belonging to G4. A score is computed based on the number of correct rankings of food healthiness within each of the four categories. If the individual correctly ranks the items according to Nova, 2 points are added up to the respective category (for example, G1>G3>G4); if the ranking between two food items is correct, but the other is not correct, only 1 point is given (for example, G1>G4>G3). Equal ratings between two groups (for example, G3=G4) also do not earn points for the total score; if the ranking of all food items according to healthiness is incorrect (e.g., G4>G3>G1), no points are awarded. Thus, the knowledge score ranges from 0 to 8 (with a maximum of 2 points per category) (7).

The Brazilian version, named Nova-Conhecimento, was developed with the original tool’s authors’ awareness, maintaining the same basic structure. Initially, a set of potential images to compose the tool was created. Thus, the frequency of food consumption in the country, according to food consumption frequency data from the 2017-2018 Brazilian Household Budget Survey (8) and the availability of items from all three Nova classification groups (G1, G3, and G4) within each category in the FoodProK were considered. Representative images of each item were searched for in a free image bank or obtained through authorial photographs. Brand names on industrialized food labels were removed, and in some cases, the original names were replaced with fictitious names created by the researchers. Industrialized food products were accompanied by standardized ingredient lists and nutritional facts labels in accordance with the food labeling regulations in effect until 2022 (9). Although new labeling regulations Industrialized food products were introduced after this data (10), the previous format was adopted, as it was the one available on most food labels in Brazil, as well as it was more familiar to consumers at the time of the study. This set of possible images was reviewed by a panel of 24 experts in the Nova classification, food labeling or the development and validation of instruments in the field of food and nutrition. Using a remote form, the experts assigned scores from 1 to 4 for relevance, clarity and appropriateness of each image. The 12 images highest-rated images were selected for inclusion in the tool, which underwent three validation stages.

The validation study was conducted in three stages (pre-testing, discriminant validation, and convergent validation) with three distinct participant groups, all recruited through convenience sampling.

The pre-testing phase involved adults recruited via the research group’s social networks, aiming to include individuals with heterogeneous sociodemographic characteristics. To define the sufficient number of contributions for this stage, a purposive approach was adopted, whereby responses were collected until comments and suggestions began to repeat without introducing new insights (11).

In the discriminant validation phase, the performance on the Nova-Conhecimento tool was compared between two groups: undergraduate students in their 4th and 5th years of nutrition studies (reference group) and undergraduate students in education-related fields (comparison group), both from the Universidade de São Paulo (USP). Participants were recruited in the classroom, with authorization from the respective professors. The required sample size was calculated based on the mean and standard deviation values of scores obtained by nutritionists in the development and evaluation study of the original FoodProK instrument (mean 7.0 and standard deviation 0.8) (7), a hypothetical standard deviation of 1.2 (50% higher than that of the reference group) was used for the calculation. To detect even a small difference of 0.5 points between groups with statistical significance, with a test power of 0.80 and an alpha of 0.05, at least 76 individuals with complete responses were estimated to be necessary for each group.

In the convergent validation phase, the association between knowledge scores and usual consumption of ultra-processed foods was tested. A random subsample of participants from the NutriNet-Brasil cohort study (n=1,245) was recruited for this purpose. NutriNet-Brasil is an open, fully online cohort conducted by the Center for Epidemiological Research on Nutrition and Health of the USP, comprising adults residing in Brazil. The subsample was stratified by sex, education level, and region. Additional details about the recruitment process for this subsample are provided in another publication (12).

In all phases, participants were asked to provide sociodemographic information and respond to the Nova-Conhecimento tool through an online form. During the pre-testing phase, which aimed to identify potential difficulties faced by the target population, participants also answered open-ended and multiple-choice questions regarding the clarity of the instructions for completing the questionnaire and any challenges encountered with the tool (13). For the convergent validation phase, the consumption of ultra-processed foods was estimated using data from up to three 24-hour dietary recalls, completed by participants as part of the NutriNet -Brazil cohort, collected over a one-year period (71.6% of individuals completed three surveys; 17.8% completed two; and 10.5% completed only one). These surveys were collected through a self-administered 24-hour dietary recall questionnaire that automatically generates estimates of the proportion of each food group from the Nova classification regarding the total caloric intake (14).

The data obtained during the pre-testing phase were qualitatively analysed to verify the need for final adjustments to the tool. The characterization of the participants for each phase was performed using absolute and relative frequency measures for categorical variables and mean and standard deviation for the age variable (continuous variable). Chi- square and Student’s t test were applied to compare the groups in the discriminant validation phase, in order to verify group comparability. This phase aimed to assess whether the tool’s score could discriminate between groups expected to perform differently on the evaluated construct (15). In this case, it was expected that nutrition students would show higher knowledge scores than education-field students. The scores obtained by each group were compared using the Mann-Whitney test, a non-parametric test for comparing means between independent samples, taking into account the asymmetric distribution of the score and considering a significance level of 5%. The convergent validation phase aimed to verify whether the new measure was associated with other variables theoretically correlated with the construct evaluated (15). In this study, the association between knowledge scores and the consumption of ultra-processed foods was acessed considering previous evidence on the correlation between studies that have already shown a correlation between food and nutrition knowledge and diet quality (16). The usual consumption of ultra-processed foods was calculated using the multiple source method, which employs repeated measures from at least one subsample of the total sample of individuals, to estimate the intra-individual variation in food consumption and remove it from the total variation (17). The association between knowledge scores (independent variable) and the usual consumption of ultra-processed foods (dependent variable) was tested using linear regression adjusted for sociodemographic characteristics. All analyses were performed using the RStudio software, version 3.6.

## Results

Initially, 40 potential food items and images were selected, grouped according to the four categories intended for the instrument – fruits, meats, cereals and dairy products.

The groups most highly rated by the experts within each category, along with the respective items to represent each group in the Nova classification, were as follows: banana-derived items (G1: fresh banana; G3: Banana sweet with added sugar; G4: banana-flavored cereal bar); beef-derived items (G1: fresh beef; G3: dried beef with added salt; G4: frozen beef meatballs); corn-derived items (G1: fresh corn on the cob; G3: canned corn; G4: corn-flavored sliced bread); and, finally, items dairy-derived items (G1: pasteurized whole cow’s milk; G3: Minas cheese; G4: strawberry-flavored dairy beverage). Adjustments to the definition and formatting of the images were also made based on the judges’ suggestions.

**Figure 1:**
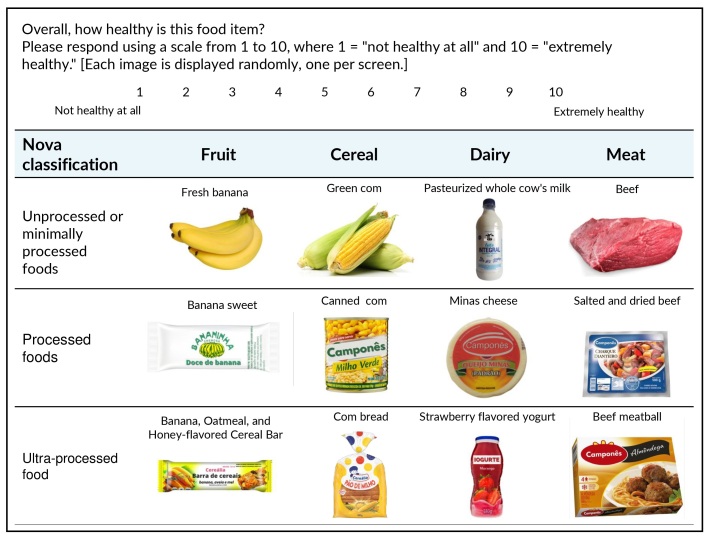
Completion instructions and images of food items comprising the Nova-Conhecimento tool

A total of twenty-four individuals participated in the pre-test. They were 75% female, 37.8 years old in average, 66.7 from the Sudest region and 66.6% had completed higher education. The respondents reported no difficulties understanding the images or instructions for completing the questionnaire; therefore, no changes were made after this stage. The version of the instrument submitted to the subsequent validation stages (discriminant and convergent) is shown in Figure 1. All complete images, including ingredient lists and nutritional facts, are available as supplementary material.

Seventy-six nutrition students and ninety-nine education students participated in the discriminant validity test. The mean age of participants was similar in both groups: 24.5 years old among nutrition students, with standard deviation (SD)=4.8, and 24.4 years old among undergraduate students, with SD=8.2 (p-value 0.920). Other sociodemographic characteristics are described in Table 1. In both groups, the majority of participants were female and individuals with incomplete higher education, with similar distributions of these characteristics between the groups. All nutrition students reported being familiar with the Guidelines, while the majority of education students reported they were unfamiliar with the document (67.7%).

**Table 1:** Sociodemographic characteristics of students participating in the discriminant validation phase. São Paulo, Brazil, 2022 (n=175)

Characteristic	Nutrition	Education Degrees	p-value
Sex			0.064
Male	11	27	
Female	65	72	
**Education level**			0.121
High school	15	31	
Higher education	61	68	
**Previous knowledge about the Guidelines**			<0.001
Yes	76	32	
No	-	67	
Total	76	99	-

In all categories, as well as in the total score, nutrition students scored higher than the education students, with statistically significant differences (Table 2).

**Table 2:** Mean and standard deviation (SD) of the total knowledge score and scores by food category according to the undergraduate course of the participants in the discriminant validation phase. São Paulo, Brazil, 2022 (n=175)

	Nutrition	Education Degrees	
Food category	Mean (SD)	Mean (SD)	p-value
Banana	1.8 (0.4)	1.4 (0.5)	<0.001
Meat	1.5 (0.5)	1.4 (0.6)	0.023
Dairy	1.6 (0.5)	1.1 (0.6)	<0.001
Corn	1.7 (0.5)	1.5 (0.5)	<0.001
Total	6.7 (1.1)	5.3 (1.2)	<0.001

In the convergent validity test, the 1,245 participants from the NutriNet-Brazil study who completed the instrument were, on average, 40.2 (SD=13.6) years old, 54.5% were female, and approximately 70% had completed high school (Table 3). The average knowledge score in this group was 5.6 (SD=1.2).

**Table 3:** Sociodemographic characteristics of the subsample of participants from the NutriNet Brasil cohort included in the convergent validation phase, Brazil, 2022 (n=1,245)

Variables	n (%)
Sex	
Male	567 (45.5)
Female	678 (54.5)
**Age group** (years)	
18-29	311 (25.0)
30-39	348 (28.0)
40-59	433 (34.8)
≥60	153 (12.3)
Region	
North	97 (7.8)
Northeast	304 (24.4)
Midwest	131 (10.5)
Southeast	496 (39.8)
South	217 (17.4)
**Education level**	
Incomplete high school	44 (3.5)
High school	823 (66.1)
Higher education	378 (30.4)
Total	1,245 (100.0)

Ultra-processed foods accounted for an average of 21,6% (SD=9.1) of the total usual calorie intake among individuals. Each 1-point increase in the knowledge score was associated with a reduction of 1.03 percentage points in the usual participation of ultra-processed foods in the diet (p-value<0.001), regardless of the sex, age and education level of the participants (Table 4).

**Table 4:** Knowledge score and standard deviation (SD) and regression coefficient (β) for the usual consumption of ultra-processed foods in the subsample of participants from the NutriNet Brazil Cohort included in the convergent validation phase. Brazil, 2022 (n=1,245)

Food category	Mean (SD)	Β	p-value
Banana	1.4 (0.5)	-1.66	0.003
Meat	1.5 (0.5)	-0.78	0.140
Dairy	1.3 (0.5)	-1.66	0.003
Corn	1.4 (0.5)	-1.85	<0.001
Total	5.6 (1.2)	-1.03	<0.001

## DISCUSSION

This study aimed to adapt and validate a tool for assessing food knowledge based on the level of food processing, specifically for the Brazilian context. Named Nova-Conhecimento, this tool is expected to be useful for assessing the implementation of the Guidelines, as it is based on the perception of the healthfulness of unprocessed and minimally processed foods, processed foods and ultra-processed foods, which constitute the cornerstone of the document’s golden rule. The set of food items for composing the Nova-Conhecimento tool was defined based on data from a national dietary survey and the evaluation of a panel of experts. The tool underwent pre-testing with laypeople, proving to be easy to understand by the general public, and showed good evidence of discriminant and convergent validity.

The results indicate that the Nova-Conhecimento tool has discriminatory power to capture differences in food knowledge levels among individuals, according to food processing level. It was expected that individuals already exposed to the Guidelines, such as nutrition students, would perform better on the tool, a finding corroborated by the literature. Another study conducted in Brazil with nutrition students showed that graduates were better able to correctly classify food items according to the Nova classification, compared to those in early stages of the course (18). Another study, which also validated a tool for assessing nutrition knowledge, but aimed at primary health care professionals, compared the performance of nutritionists with that of other health professionals, and similarly found higher scores among specialists (19).

Findings from the convergent validity reinforce that the score generated by the Nova-Conhecimento tool reflects the ability to distinguish foods as more or less healthful, considering their processing level. The inverse association between knowledge scores and the consumption of ultra-processed foods aligns with other studies, which indicate that greater food knowledge is associated with diet quality (16). A common limitation in studies investigating this relationship is the mismatch between the aspects of diet assessed in terms of knowledge and consumption, which is a strength of this study, since the food processing paradigm was considered in both cases (16).

This study has some limitations that are worth highlighting. In the pre-test, the sample was not very diverse, both in terms of distribution by education level and geographic regions of the country. However, the convergent validation included individuals from all regions of the country and with lower levels of education, mitigating this limitation, as the association with the consumption of ultra-processed foods remained consistent, even when adjusted for these characteristics.

A key limitation of the Nova-Conhecimento tool pertains to the outdated labeling of the images used, which does not reflect current legislation. In 2020, Brazil enacted a revision of food labeling regulations (10), introducing changes to the nutritional information tables and requiring warning labels on products with high levels of fat, sugar and sodium. As for the nutritional table, the current version allows for greater comparability regarding the nutritional composition of similar products, as it presents a column with information per 100g of food, in addition to requiring the inclusion of added sugar information. However, these changes are not expected to affect the Nova-Conhecimento tool’s score, as it is based on comparisons between products with different levels of processing. Furthermore, the core element of the label for identifying an ultra-processed food is its ingredient list, which remained unchanged (20).

As for warning labels, it has been demonstrated that they indeed influence the perception of the product healthfulness, with most products receiving such labels being ultra-processed (21,22). However, it is likely that including the labels in the images of this study would not change these results, as both processed and ultra-processed products would receive labels. According to the new rules (10), in the banana category, both the banana sweet (G3) and the cereal bar (G4) would receive the “high in sugar” label. In the meat category, the dried beef (G3) would receive the sodium label, while meatballs (G4) would receive a saturated fat label. In the corn and dairy categories, no product would receive warnings. Although it is believed that this factor does not invalidate the results of this study, researchers using the tool in the future may choose to update the table and include warning labels in the product images.

The potential uses and applications of the Nova-Conhecimento tool are diverse, given its simplicity and potential application in both paper and digital format. It can be used not only in research aimed at identifying the level of dissemination of the Guidelines at local or national levels, but also to evaluate the effect of educational interventions based on the Nova classification to improve individuals’ knowledge. Future studies could test its validity for other population groups, such as adolescents.

In conclusion, the proposed tool, named Nova-Conhecimento, provides evidence of validity for measuring food knowledge based on the Nova classification, and can serve as an important instrument for evaluating the dissemination of the *Dietary Guidelines for the Brazilian Population* and food and nutrition education interventions based on it.
