# Supplementary material for: Adaptation and validation of a tool for assessing food knowledge based on the Nova classification for the Brazilian context
Source: Epidemiol Serv Saude. 2025 May 2;34:e20240335. doi: 10.1590/S2237-96222024v34e20240335.en (PMC12052328; doi:10.1590/S2237-96222024v34e20240335.en)
Supplement: Translation

# Translation: Adaptação e validação para o contexto brasileiro de ferramenta para avaliação do conhecimento em alimentação baseada na classificação Nova

**DOI:** 10.1590/S2237-96222024v34e20240335.pt

**Authors:** Kamila Tiemann Gabe, Gilberto Bassetto, Patricia Constante Jaime

**Affiliations:** 1 Universidade de São Paulo, Núcleo de Pesquisas Epidemiológicas em Nutrição e Saúde, São Paulo, SP, Brasil; 2 Universidade de São Paulo, Faculdade de Saúde Pública, Departamento de Nutrição, São Paulo, SP, Brasil

**Keywords:** Guias Alimentares, Educação Alimentar e Nutricional, Letramento em Saúde, Programas e Políticas de Nutrição e Alimentação, Estudos de Validação

## Abstract

**Objetivos:**

Adaptar e validar, para o contexto brasileiro, uma ferramenta para mensurar o nível de conhecimento em alimentação segundo a classificação Nova.

**Métodos:**

Adaptou-se para o Brasil uma ferramenta desenvolvida por pesquisadores canadenses em que notas de saudabilidade são atribuídas a 12 imagens de alimentos com diferentes níveis de processamento industrial segundo a classificação Nova – in natura e minimamente processados, processados e ultraprocessados. Escores são computados com base na comparação entre notas atribuídas a alimentos de diferentes grupos, podendo variar de 0 a 8. A versão brasileira, denominada Nova-Conhecimento, foi avaliada por especialistas e submetida a pré-testes com potenciais usuários da ferramenta. Avaliou-se sua validade discriminante, comparando-se escores de estudantes de nutrição (n=76) e licenciaturas (n=99). A validade convergente foi avaliada testando-se a associação entre o escore de conhecimento e o consumo de ultraprocessados em uma subamostra da coorte NutriNet Brasil (n=1.245).

**Resultados:**

Estudantes de nutrição apresentaram escore superior aos de licenciatura (6,7 vs. 5,3; p-valor<0,001). Cada ponto de escore esteve associado à redução de 1,03 ponto percentual na participação de ultraprocessados na dieta (p-valor<0,001).

**Conclusão:**

A Nova-Conhecimento mostrou-se válida e pode contribuir para ações de vigilância alimentar e nutricional baseadas no *Guia Alimentar para a População Brasileira*.

## Introdução

Uma das funções primordiais de um guia alimentar é servir como fonte de informação oficial e confiável sobre alimentação saudável para a população de cada país (1). A segunda edição do *Guia Alimentar para a População Brasileira*, publicada em 2014 pelo Ministério da Saúde, propôs, pela primeira vez, recomendações inteiramente baseadas no nível de processamento dos alimentos, resumidas na regra de ouro “prefira sempre alimentos in natura e minimamente processados e suas preparações culinárias a alimentos ultraprocessados” (2).

Essa mensagem central foi baseada em dados que apontavam tendência de substituição pela população brasileira do consumo de alimentos tradicionais, como arroz e feijão, pelo consumo de alimentos ultraprocessados (3), e tem se revelado cada vez mais pertinente, levando em conta o crescente conjunto de evidências sobre os impactos negativos à saúde do maior consumo desses alimentos (4). Para que a redução do consumo de ultraprocessados seja alcançada, promover o conhecimento da população para identificar esses alimentos e reconhecê-los como não saudáveis é fundamental para que indivíduos tenham autonomia para realizar escolhas alimentares saudáveis. 

Desde a sua publicação, diversas ações foram empreendidas para disseminar as recomendações do Guia, como a publicação de cartilhas, vídeos e fôlderes, a formação de profissionais da saúde e a inclusão de mensagens em livros didáticos (5). Apesar disso, o conhecimento da população brasileira para distinguir alimentos como mais ou menos saudáveis, considerando o seu grau de processamento, ainda é pouco estudado, limitando a possibilidade de análise do impacto do Guia. Isso, em parte, se deve à ausência de ferramentas validadas que permitam essa mensuração.

Um estudo que revisou instrumentos de promoção da alimentação saudável e de avaliação da adesão ao Guia encontrou duas ferramentas para avaliar o conhecimento de indivíduos segundo suas recomendações já desenvolvidas no Brasil. Entre essas duas, uma é voltada para profissionais da saúde e a outra para crianças, sendo que nenhuma delas é focada em medir a habilidade de reconhecer alimentos ultraprocessados (6). Recentemente, pesquisadores canadenses desenvolveram uma ferramenta para medir o conhecimento sobre alimentação baseada na Nova, o sistema de classificação de alimentos segundo grau de processamento que introduziu o conceito de alimentos ultraprocessados, e que é adotado no Guia (7). 

A ferramenta *food processing knowledge* (FoodProK) é empregada para avaliar a capacidade de indivíduos em reconhecer alimentos ultraprocessados como menos saudáveis do que alimentos das outras categorias da classificação Nova, por meio de um sistema de atribuição de notas de saudabilidade a diversos alimentos (7). Um escore é computado com base na comparação de notas atribuídas aos alimentos, segundo grupos da classificação Nova. O objetivo deste estudo foi adaptar e validar essa ferramenta para o contexto brasileiro, a fim de mensurar o nível de conhecimento em alimentação segundo a classificação Nova. 

## Método

Foi realizada a adaptação e validação, para o contexto brasileiro, de uma ferramenta para avaliar o conhecimento em alimentação, segundo o grau de processamento dos alimentos.

Na FoodProK, ferramenta utilizada como modelo para o desenvolvimento da ferramenta brasileira, os respondentes são solicitados a avaliar, em uma escala de 1 a 10, o quão saudável consideram uma sequência de 12 itens alimentares, distribuídos em quatro categorias – frutas, carnes, laticínios e cereais. Os alimentos são apresentados individualmente em ordem aleatória; itens embalados são acompanhados de tabela nutricional e lista de ingredientes. Dentro de cada uma das quatro categorias, um alimento pertencendo a cada um dos seguintes grupos alimentares da classificação Nova é apresentado: alimentos in natura e minimamente processados (G1), alimentos processados (G3) e alimentos ultraprocessados (G4). Os ingredientes culinários processados (G2) não são incluídos na ferramenta, pois não costumam ser consumidos sozinhos, mas como parte de preparações culinárias. Para mensuração do nível do conhecimento, é esperado que, dentro de cada categoria de alimentos, o indivíduo avalie o alimento pertencente ao G1 com maior nota; o do G3, com nota intermediária; e o do G4, com menor nota. Um escore é computado com base no número de acertos na ordem de saudabilidade dos alimentos, dentro de cada uma das quatro categorias. Se o indivíduo atribuir pontos na ordem correta de acordo com a Nova, 2 pontos são computados na respectiva categoria (por exemplo, G1>G3>G4); caso a ordem entre dois alimentos esteja correta, mas a outra não, somente 1 ponto é conferido (por exemplo, G1>G4>G3). Respostas iguais entre dois grupos (por exemplo, G3=G4) também não geram pontos para o escore total; se a posição de todos os alimentos na ordem de saudabilidade estiver errada (por exemplo, G4>G3>G1), nenhum ponto é computado. Dessa forma, o escore de conhecimento pode variar de 0 a 8 (máximo de 2 pontos por categoria) (7). 

A Nova-Conhecimento, nome atribuído à versão adaptada para o Brasil, foi elaborada sob ciência dos autores da ferramenta original, mantendo-se a mesma estrutura básica. Primeiramente, elaborou-se um conjunto inicial com potenciais imagens para compor a ferramenta. Para isso, considerou-se tanto a frequência de consumo dos alimentos no país, de acordo com os dados de consumo alimentar da Pesquisa de Orçamentos Familiares de 2017-2018 (8), como a existência, em cada categoria, de itens dos três grupos da classificação Nova adotados na FoodProk (G1, G3 e G4). Imagens representativas de cada item foram buscadas em banco de imagens gratuito ou obtidas por meio de fotografias autorais. Removeu-se, dos rótulos de alimentos industrializados, os nomes de marcas, sendo que, em algumas imagens, os nomes originais foram substituídos por nomes fictícios criados pelos pesquisadores. Para alimentos industrializados, as imagens foram acompanhadas da lista de ingredientes e de tabelas nutricionais, que foram padronizadas em conformidade com as normas de rotulagem de alimentos vigentes até o ano de 2022 (9). Embora novas normas para a rotulagem de alimentos tenham entrado em vigência após esse ano (10), optou-se por manter o formato anterior, pois, até o momento da condução do estudo, aquele era o que se apresentava disponível na maioria dos rótulos de alimentos no Brasil, sendo mais familiar para os consumidores. Este conjunto de possíveis imagens foi submetido à avaliação de um painel de juízes com 24 especialistas na classificação Nova, em rotulagem de alimentos ou em desenvolvimento e validação de instrumentos na área de alimentação e nutrição. De forma remota, os especialistas preencheram um formulário por meio do qual atribuíram notas de 1 a 4 para relevância, clareza e pertinência de cada imagem. As 12 imagens mais bem avaliadas pelos especialistas foram selecionadas para compor o instrumento, que foi submetido a três etapas de validação.

O estudo de validação da ferramenta foi realizado em três etapas (pré-teste, validação discriminante e validação convergente), com três grupos distintos de participantes, todos obtidos por meio de amostragem de conveniência. 

Da etapa de pré-testes, participaram adultos recrutados por meio das redes sociais do grupo de pesquisa, buscando-se alcançar indivíduos com características sociodemográficas heterogêneas. Para definição do número de contribuições suficientes nessa etapa, foi adotada abordagem propositiva, ou seja, as respostas foram recrutadas até o ponto em que os comentários e sugestões começassem a se repetir, sem que aparecesse algo novo (11). 

Na etapa de validação discriminante, comparou-se o desempenho na Nova-Conhecimento de dois grupos de indivíduos: estudantes do 4º e 5º ano do curso de graduação em nutrição (grupo de referência) e estudantes de cursos de graduação em licenciaturas (grupo de comparação), ambos da Universidade de São Paulo (USP). O recrutamento dos participantes foi realizado em sala de aula, mediante a autorização dos professores responsáveis pelas turmas. Calculou-se o número amostral necessário com base nos valores de média e desvio-padrão do escore obtido por nutricionistas no estudo de desenvolvimento e avaliação da versão original do instrumento FoodProK (média 7,0 e desvio-padrão 0,8) (7). Assumindo-se que a variabilidade de escore entre não especialistas poderia ser maior, utilizou-se, no cálculo amostral, um valor hipotético de desvio-padrão de 1,2 (50% a mais do que o do grupo de referência). Para que uma diferença de pelo menos 0,5 ponto de escore entre os grupos pudesse ter significância estatística, para um poder de teste de 0,80 e alfa de 0,05, estimou-se que seriam necessários pelo menos 76 indivíduos com respostas completas em cada grupo. 

Na etapa de validação convergente, testou-se a associação entre o escore de conhecimento e o consumo usual de alimentos ultraprocessados. Para isso, recrutou-se uma subamostra aleatória de participantes do estudo de coorte NutriNet-Brasil (n=1.245), uma coorte aberta e totalmente online conduzida pelo Núcleo de Pesquisas Epidemiológicas em Nutrição em Saúde da USP, da qual participam adultos que residem no Brasil. O sorteio e recrutamento dessa subamostra foi baseado em cotas de sexo, escolaridade e região. Mais detalhes sobre o recrutamento dessa amostra podem ser conferidos em outra publicação (12).

Em todas as etapas, os participantes foram solicitados a fornecer informações sociodemográficas e, em seguida, a responder à Nova-Conhecimento por meio de um formulário online. Na etapa de pré-teste, que teve como finalidade identificar potenciais dificuldades por parte do público ao qual se destina, os indivíduos também responderam a questões abertas e de múltipla escolha a respeito da clareza das instruções para o preenchimento e de eventuais dificuldades encontradas com a ferramenta (13). Para a etapa de validação convergente, o consumo de alimentos ultraprocessados foi estimado utilizando-se dados de até três recordatórios alimentares de 24 horas, respondidos pelos indivíduos como parte da coorte NutriNet-Brasil e coletados ao longo de um ano (71,6% dos indivíduos tinham três inquéritos preenchidos; 17,8% tinham dois; e 10,5% tinham apenas um). Esses inquéritos foram coletados por meio de um questionário recordatório alimentar de 24 horas autoaplicável que gera automaticamente estimativas de participação de cada grupo alimentar da classificação Nova no total de calorias consumidas (14).

Os dados obtidos na etapa de pré-teste foram avaliados qualitativamente e utilizados para verificação da necessidade da realização de ajustes finais na ferramenta. A caracterização dos participantes de cada etapa foi realizada por meio de medidas de frequência absoluta e relativa para as variáveis categóricas e média e desvio-padrão para a variável idade (variável contínua). Testes qui-quadrado e t de Student foram aplicados para comparar os grupos da etapa de validação discriminante, a fim de se verificar a comparabilidade dos grupos. Essa etapa teve como objetivo verificar se o escore gerado pela ferramenta é capaz de discriminar grupos dos quais já se esperaria desempenho diferente em relação ao constructo avaliado (15). Neste caso, era esperado que os estudantes de nutrição apresentassem escores de conhecimento mais elevados do que os estudantes de licenciatura. Foram comparados os escores obtidos por cada grupo por meio do teste de Mann-Whitney, teste não-paramétrico de comparação de médias entre amostras independentes, levando-se em conta a distribuição assimétrica do escore, considerando-se o nível de significância de 5%. A etapa de validação convergente teve como objetivo verificar se a nova medida se associa com outras variáveis teoricamente correlacionadas ao constructo avaliado (15). Neste estudo, avaliou-se a associação entre o escore de conhecimento e o consumo de alimentos ultraprocessados, considerando-se estudos que já mostraram que há correlação entre conhecimento em alimentação e nutrição e qualidade da alimentação (16). Calculou-se o consumo usual de alimentos ultraprocessados por meio do método *multiple source method*, que utiliza medidas repetidas coletadas em pelo menos uma subamostra do total de indivíduos da amostra, para estimar a variação intraindividual do consumo alimentar e removê-la da variação total (17). A associação entre o escore de conhecimento (variável independente) e o consumo usual de alimentos ultraprocessados (variável dependente) foi testada por meio de regressão linear ajustada para características sociodemográficas. Todas as análises foram realizadas com utilização do software RStudio versão 3.6.

## Resultados

Inicialmente, foram selecionados 40 possíveis alimentos e imagens, agrupados de acordo com as quatro categorias pretendidas para o instrumento – frutas, carnes, cereais e laticínios. 

Os agrupamentos mais bem avaliados pelos especialistas dentro de cada categoria, bem como os respectivos itens para representar cada grupo da Nova, foram: itens derivados da banana (grupo 1: banana in natura; grupo 3: doce de banana com adição de açúcar; grupo 4: barra de cereal sabor banana); itens derivados de carne bovina (G1: carne bovina fresca; G3: carne seca com adição de sal; G4: almôndega bovina congelada); itens derivados do milho (G1: espiga de milho in natura; G3: milho enlatado; G4: pão de forma sabor milho); e, por fim, itens derivados de laticínios (G1: leite de vaca integral; G3: queijo Minas; G4: bebida láctea sabor morango). Ajustes na definição e formatação das imagens também foram realizados por sugestão dos juízes. 

Participaram do pré-teste 24 pessoas, com média de 37,8 anos, sendo 75,0% do sexo feminino, a maioria com ensino superior completo (66,6%) e a maioria da região Sudeste do Brasil (66,7%). Os respondentes não reportaram dificuldades em compreender as imagens ou instruções para preenchimento, portanto nenhuma alteração foi realizada após esta etapa. A versão do instrumento submetida às etapas de validação subsequentes (discriminante e convergente) é apresentada na Figura 1. Todas as imagens completas, com listas de ingredientes e tabelas nutricionais, estão disponíveis como material suplementar. 

**Figura 1:** Instruções de preenchimento e imagens de alimentos que compõem a ferramenta Nova-Conhecimento

Participaram do teste de validade discriminante 76 estudantes de graduação em nutrição e 99 de graduação em licenciaturas. A média de idade dos participantes foi similar nos dois grupos: 24,5 anos entre os estudantes de nutrição, com desvio padrão (DP)=4,8, e 24,4 anos entre os estudantes de licenciatura, com DP=8,2 (p-valor 0,920). As demais características sociodemográficas são descritas na Tabela 1. Nos dois grupos, a maioria dos participantes eram mulheres e pessoas com ensino superior incompleto, sendo as distribuições dessas características similares nos dois grupos. Todos os estudantes de nutrição afirmaram conhecer o Guia, enquanto a maioria dos estudantes de licenciaturas referiu não conhecer o documento (67,7%).

**Tabela 1:** Características sociodemográficas dos estudantes participantes da etapa de validação discriminante. São Paulo, 2022 (n=175)

Característica	Nutrição	Licenciaturas	p-valor
Sexo			0,064
Masculino	11	27	
Feminino	65	72	
Escolaridade			0,121
Médio	15	31	
Superior	61	68	
**Conhecimento sobre o Guia**			<0,001
Sim	76	32	
Não	-	67	
Total	76	99	-

Em todas as categorias de alimentos, assim como no escore total, os estudantes de nutrição obtiveram pontuações superiores às dos estudantes de licenciaturas, com diferenças estatisticamente significativas (Tabela 2). 

**Tabela 2:** Média e desvio padrão (DP) do escore total e dos escores por categorias de alimentos de acordo com o curso de graduação dos participantes da etapa de validação discriminante. São Paulo, 2022 (n=175)

	Nutrição	Licenciaturas	
Categoria de alimento	Média (DP)	Média (DP)	p-valor
Banana	1,8 (0,4)	1,4 (0,5)	<0,001
Carne	1,5 (0,5)	1,4 (0,6)	0,023
Laticínio	1,6 (0,5)	1,1 (0,6)	<0,001
Milho	1,7 (0,5)	1,5 (0,5)	<0,001
Total	6,7 (1,1)	5,3 (1,2)	<0,001

No teste de validade convergente, os 1.245 participantes do estudo NutriNet-Brasil que preencheram o instrumento tinham, em média, 40,2 (DP=13,6) anos, sendo 54,5% mulheres, e aproximadamente 70% com escolaridade até o ensino médio completo (Tabela 3). A média do escore de conhecimento segundo o grau de processamento dos alimentos, neste grupo, foi 5,6 (DP=1,2).

**Tabela 3:** Características sociodemográficas da subamostra de participantes da coorte NutriNet Brasil incluídos na etapa de validação convergente, Brasil, 2022 (n=1.245)

Variáveis	n (%)
Sexo	
Masculino	567 (45,5)
Feminino	678 (54,5)
**Faixa etária** (anos)	
18-29	311 (25,0)
30-39	348 (28,0)
40-59	433 (34,8)
≥60	153 (12,3)
Região	
Norte	97 (7,8)
Nordeste	304 (24,4)
Centro-Oeste	131 (10,5)
Sudeste	496 (39,8)
Sul	217 (17,4)
Escolaridade	
Médio incompleto	44 (3,5)
Médio	823 (66,1)
Superior	378 (30,4)
Total	1.245 (100,0)

Os alimentos ultraprocessados corresponderam, em média, a 21,6% (DP=9,1) do total de calorias consumidas usualmente pelos indivíduos. Cada acréscimo de 1 ponto de escore de conhecimento esteve associado à redução de 1,03 ponto percentual da participação usual de alimentos ultraprocessados na dieta (p-valor<0,001), independentemente do sexo, da idade e da escolaridade dos participantes (Tabela 4).

**Tabela 4:** Escore de conhecimento e desvio padrão (DP) e coeficiente de regressão (β) do consumo usual de alimentos ultraprocessados na subamostra de participantes da Coorte NutriNet Brasil incluída na etapa de validação convergente. Brasil, 2022 (n=1.245)

Categoria de alimento	Média (DP)	β	p-valor
Banana	1,4 (0,5)	-1,66	0,003
Carne	1,5 (0,5)	-0,78	0,140
Laticínio	1,3 (0,5)	-1,66	0,003
Milho	1,4 (0,5)	-1,85	<0,001
Total	5,6 (1,2)	-1,03	<0,001

## Discussão

Este estudo propôs a adaptação e validação, para o contexto brasileiro, de uma ferramenta para avaliação do conhecimento em alimentação segundo o nível de processamento dos alimentos. Denominada Nova-Conhecimento, espera-se que esta ferramenta possa ser útil para avaliação da implementação do Guia, já que é baseada na percepção da saudabilidade de alimentos in natura e minimamente processados, processados e ultraprocessados, que constituem a base da regra de ouro do documento. O conjunto de alimentos para composição da ferramenta Nova-Conhecimento foi definido com base nos dados de um inquérito nacional de consumo alimentar e na avaliação de um grupo de especialistas. A ferramenta passou por pré-teste com pessoas leigas, mostrando-se de fácil compreensão pelo público em geral, e apresentou boas evidências de validade discriminante e convergente.

Os resultados mostram que a ferramenta Nova-Conhecimento tem poder discriminatório para capturar diferenças no nível de conhecimento em alimentação entre indivíduos, segundo o nível de processamento. Isso porque era esperado que indivíduos já expostos ao Guia, no caso, os estudantes de nutrição, obtivessem melhor resultado na ferramenta, o que é corroborado pela literatura. Uma pesquisa realizada no Brasil com estudantes de graduação em nutrição mostrou que egressos possuíam maior habilidade de classificar corretamente alimentos segundo a classificação Nova, em comparação a ingressantes (18). Outro estudo, também de validação de uma ferramenta para avaliar conhecimento sobre nutrição, mas voltada para profissionais da atenção primária à saúde, comparou o desempenho de nutricionistas com a de demais profissionais, também encontrando maiores escores entre os especialistas (19).

Os achados da etapa de validação convergente reforçam que o escore gerado pela ferramenta Nova-Conhecimento reflete a habilidade de distinguir alimentos como mais ou menos saudáveis, considerando-se o nível de processamento. A associação inversa entre o escore de conhecimento e o consumo de alimentos ultraprocessados está em congruência com outros estudos, os quais apontam que o maior conhecimento em alimentação se associa com a qualidade da alimentação (16). Uma limitação comum de estudos investigando essa relação é o desalinhamento entre os aspectos de alimentação avaliados quanto ao conhecimento e quanto ao consumo, o que é um ponto forte deste estudo, já que o paradigma do processamento de alimentos foi considerado nos dois casos (16).

Este estudo apresentou algumas limitações que merecem ser destacadas. No pré-teste, a amostra se apresentou pouco diversa, tanto em termos de distribuição em níveis de escolaridade quanto nas regiões geográficas do país. Contudo, a etapa de validação convergente incluiu pessoas de todas as regiões do país e com menores níveis de escolaridade, o que minimiza essa limitação, já que a associação com o consumo de ultraprocessados foi consistente, mesmo quando ajustado para essas características.

Uma limitação-chave da Nova-Conhecimento refere-se à desatualização dos rótulos utilizados nas imagens quanto à legislação atual. Em 2020, foi promulgada no país uma revisão das normas para rotulagem de alimentos (10), com alterações na tabela de informações nutricionais e inclusão de selos de advertência em produtos com altos níveis de gordura, açúcar e sódio. Quanto à tabela nutricional, a versão vigente permite maior comparabilidade quanto à composição nutricional de produtos similares, pois apresenta uma coluna com informação para 100g de alimento, além de obrigar a inclusão da informação sobre açúcar adicionado. Contudo, no caso da Nova-Conhecimento, não é esperado que a mudança na tabela altere o escore de conhecimento, já que este é baseado na comparação entre diferentes produtos, com diferentes níveis de processamento. Ademais, o elemento central do rótulo para identificação de um alimento ultraprocessado é a sua lista de ingredientes, que permaneceu inalterada (20).

Quanto aos selos de advertência, já foi demonstrado que de fato influenciam a percepção de saudabilidade de alimentos, e que a maioria dos produtos que recebem selos são ultraprocessados (21,22). Contudo, é provável que a inclusão dos selos nas imagens da Nova-Conhecimento não alterasse estes resultados, pois tanto produtos processados quanto ultraprocessados receberiam selos. Segundo as novas normas (10), na categoria da banana, tanto a bananinha (G3) quanto a barra de cereal (G4) receberiam o selo de “alto em açúcar”. Na categoria da carne, a carne seca (G3) receberia o selo do sódio, enquanto a almôndega (G4) receberia o selo da gordura saturada. Nas categorias do milho e do laticínio, nenhum produto receberia advertência. Embora se acredite que esse fator não invalide os resultados do presente estudo, pesquisadores que venham a utilizar a ferramenta podem optar por atualizar a tabela e introduzir o selo de advertência nas imagens apresentadas aos respondentes.

Os potenciais usos e aplicações da ferramenta Nova-Conhecimento são diversos, dada a sua simplicidade e potencial aplicação, tanto em papel quanto por meio digital. Pode ser aplicada não somente em pesquisas que visem identificar o grau de disseminação do Guia em nível local ou nacional, mas também para avaliar o efeito de intervenções educativas baseadas na classificação Nova voltadas para aumentar o conhecimento dos indivíduos. Futuros estudos poderiam testar a sua validade para outros grupos populacionais, como adolescentes.

Em conclusão, a ferramenta proposta, denominada Nova-Conhecimento, apresenta evidência de validade para medir o conhecimento em alimentação segundo a classificação Nova entre adultos, podendo servir como um importante instrumento para a avaliação da disseminação do *Guia Alimentar para a População Brasileira* e de intervenções de educação alimentar e nutricional nele baseadas.
